# A Systematic Review of the Accuracy and Reliability of Pyuria in Diagnosing Pediatric Urinary Tract Infections

**DOI:** 10.7759/cureus.79135

**Published:** 2025-02-17

**Authors:** Shams K Sameer, Mohammad J Taha, Warda A Alrubasy, Abdalah T Abozenah, Malek Y Alkiswani, Abdullah A Elhakim, Mohamed H Megali, Anas M Alshami, Abdulqadir J Nashwan, Mohammad T Abuawwad

**Affiliations:** 1 Pediatrics, Cairo University, Cairo, EGY; 2 Neurology, Cairo University, Cairo, EGY; 3 Nursing and Midwifery Research, Hamad Medical Corporation, Doha, QAT

**Keywords:** children, infection, pediatrics, pyuria, uti

## Abstract

Urinary tract infections (UTIs) are among the most common bacterial infections during childhood. Early diagnosis and prompt treatment are necessary to prevent long-term sequelae. Pyuria, a key diagnostic marker for UTIs, is defined as the presence of ≥5 white blood cells per high-power field in urine obtained by centrifugation of urine and microscopic analysis. However, there is a debate around pyuria's role in UTI diagnosis, which highlights the need for a comprehensive evaluation of its diagnostic accuracy in pediatric settings.

This study aims to evaluate the diagnostic accuracy of pyuria as a marker for UTIs in pediatric patients by analyzing the sensitivity, specificity, and various factors affecting the diagnostic performance of pyuria and the alignment of these findings with the current clinical guidelines of pediatric UTI management.

A comprehensive search was conducted through the following electronic databases: PubMed, Central, and Science Direct. The articles underwent a two-phase filtration process: first by title and abstract and second by full text, conducted by two independent reviewers. Data was extracted using a Google form (Google LLC, Mountain View, CA, USA) covering study details, participant characteristics, biomarkers, and diagnostic methods. The methodological quality of the studies was assessed using the National Institutes of Health tool, and the detailed protocol is available on PROSPERO (CRD42023399392).

Our search yielded 491 results, with 18 studies meeting the inclusion criteria. The mean age across the studies was 2.7 years, and the majority of patients were females. *Escherichia coli *was the predominant pathogen, accounting for the infection in 5,696 (61%) out of 9,628 positive cultures. The included articles reported positive pyuria in 64% of the patients with positive cultures. It was found that the type of uropathogen, urinalysis techniques, urine concentration, patient demographics, and underlying congenital anomalies are among the factors that affect the diagnostic accuracy of pyuria. A generated receiver operating characteristic (ROC) revealed a higher diagnostic performance for pyuria with an area under the curve of (area under the curve (AUC)=0.793) compared to that of nitrite (AUC=0.671). Notably, pyuria was significantly associated with *Escherichia coli* infections, although *Escherichia coli* represented the majority of cases overall. Urinalysis techniques influenced the sensitivity and specificity of pyuria. Urinalysis performed by automated methods achieved sensitivity rates of 80% and specificity rates of 90%, whereas enhanced methods showed higher sensitivity at 84% and specificity at 94%.

The absence of pyuria does not rule out the diagnosis of UTI among pediatric age groups who display symptoms of UTI. The onset of urinalysis in relation to fever onset, type of causative urinary pathogen, urine concentration, and other individual factors may lead to the absence of pyuria in the presence of true UTI. Further research is required to assess the diagnostic criteria for UTI among pediatrics and investigate the role of other biomarkers in accurately diagnosing UTI.

## Introduction and background

Urinary tract infections (UTIs) are among the most common bacterial infections in children, affecting up to 8% of infants during their first year of life, with recurrence rates reaching 30% after an initial episode of UTI, and are considered an important cause of neonatal morbidity and mortality [1,2]. During the first year of life, the incidence of UTIs in girls is 0.7% compared with 2.7% in boys [3,4]. This is more evident in the first two months of life, with an incidence of 5% in girls and 20.3% in uncircumcised boys [1]. *Escherichia coli* is the most common causative organism of UTI, accounting for over 80% of cases. Other organisms include *Enterobacter aerogenes, Klebsiella pneumoniae, Proteus mirabilis, Citrobacter, Pseudomonas aeruginosa, Enterococcus spp., and Serratia spp.* Children with underlying renal tract anomalies are subjected to infection with *Staphylococcus aureus, Staphylococcus epidermidis, Haemophilus influenzae, Streptococcus pneumoniae, Streptococcus viridians, *and *Streptococcus agalactiae* [5].

The clinical presentation of UTI among pediatrics is often heterogenous, with subtle signs and symptoms that present a diagnostic challenge to clinicians. Symptoms vary significantly among different pediatric age groups. During the first two years of life, fever of unknown origin is the most common symptom among children with UTI. Specific symptoms such as malodorous urine, dysuria, and flank pain start to become prevalent after the age of two years [5,6]. Urine culture is the gold standard method for the diagnosis of UTI. However, due to it being time-consuming, most clinicians rely on the degree of pyuria obtained from rapid dipstick urinalysis as an initial diagnosis of UTI [7]. Pyuria is the presence of (≥5 white blood cells/high-power field) white blood cells in the urine. It occurs as a result of the inflammatory response of the urinary epithelium elicited by bacterial invasion [8]. Pyuria was considered a cornerstone in diagnosing UTI [9]. Recently, however, the reliability of pyuria as a diagnostic marker for UTI in pediatric patients has been debated. Different urinary pathogens organisms have been associated with varying levels of pyuria; non-*Escherichia coli *strains such as *Enterococcus *species and *Klebsiella *species were found to cause a UTI in the absence of pyuria. Moreover, the low specificity of pyuria has been observed in children with fever from infections unrelated to the urinary tract, patients with Kawasaki disease, and cases of rigorous exercise, who were found to have pyuria without an underlying UTI [2,10,11]. The absence of pyuria during an initial urinalysis may result in delayed diagnosis and delayed antimicrobial therapy. Delayed diagnosis of UTI carries serious consequences, including renal scarring, recurrent pyelonephritis, impaired glomerular functions, and end-stage renal disease. It is noteworthy to mention that in 30% of children with underlying congenital anomalies of the kidney and urinary tract, UTI could be the first sign [4,12]. Due to the devastating consequences it carries, UTI management guidelines in children emphasize the importance of diagnosis and treatment of UTI to prevent long-term renal injury. According to the American Academy of Pediatrics, the presence of pyuria and at least 50,000 CFU/mL of a single uropathogen in an appropriately obtained specimen of urine culture is used to diagnose UTI [2]. Despite the aforementioned guidelines, cases of UTI without pyuria have been reported. Around 10% of symptomatic children with positive urine cultures can exhibit the absence of pyuria on urinalysis [13].

In this paper, we aim to investigate the significance of pyuria’s diagnostic accuracy in pediatric UTI by analyzing cases from literature where pyuria was reportedly absent in confirmed UTI cases and diving into the various factors and their mechanisms in pediatric patients that may contribute to this finding.

## Review

Methodology

Study Design

This research was conducted following the Preferred Reporting Items for Systematic Reviews and Meta-Analyses (PRISMA) guidelines, and the protocol was preregistered on PROSPERO (CRD42023399392) on March 3, 2023. The research design followed the PEO framework as follows: population (pediatrics and individuals under the age of 18), exposure (pyuria), and outcome (diagnosis of UTI). The study design was restricted to observational cohort and cross-sectional studies.

The analysis of the microbiological data among studies revealed a total of 9,286 positive urine cultures. Urine was collected using the following methods: clean catch (CC), urethral catheterization, suprapubic aspiration (SPA), and sterile urine bag (SUB). Gram-negative organisms were dominant over gram-positive organisms. The most frequently isolated pathogen among the urine cultures was *Escherichia coli*, identified in 5,696 (61%) patients, followed by *Klebsiella* species (17%), *Enterococcus* species (5.2%), *Proteus* species (5.1%), *Enterobacter* species (4.8%), *Pseudomonas* species (0.8%), *Staphylococcus saprophyticus* (0.3%), and others (4.1%). Pyuria was found in 5,965 (64%) of patients with positive urine cultures and was absent in 3,321 (36%) of patients. Among all the studies that reported it, nitrite was positive in 73%, and leukocyte esterase was positive in 0.12%.

Search Strategy­­

On February 15th, 2023, a comprehensive electronic search was conducted through the following databases: PubMed, Central, and Science Direct, for all the studies reporting suspected UTI among children under 18 years with respect to pyuria. Moreover, a manual search was carried out to ensure the identification of all relevant studies through the following approaches: (a) searching the reference list of the final included papers in our review, (b) using the "similar articles" option on PubMed, and (c) manually searching for articles on Google using the following keywords: “pyuria”, “UTI”, and “children”.

Eligibility Criteria

The included studies had to fulfill the following criteria: observational studies discussing the presence or absence of pyuria among children under the age of 18 suspected to have a UTI. Only English articles published after 2011 were included to minimize the diagnostic variability, as the American Academy of Pediatrics published the guidelines standardizing the diagnosis of pyuria in 2011 [2]. No limitations were set on the country of publication. Duplicate studies, unavailable full texts, non-original research (reviews, commentaries, guidelines, editorials, correspondence, letters to editors, etc.), and unrelated papers to this study objective were excluded.

Study Selection

Following their retrieval from the database search, the studies were imported into EndNote (Clarivate, London, United Kingdom) for duplicate removal. Then, the citations were exported to an Excel sheet (Microsoft Corporation, Redmond, WA, USA) for screening. The retrieved articles were subject to two phases of filtration. First, the titles and abstracts of the retrieved articles were screened against the predefined eligibility criteria. Then, relevant studies underwent full-text screenings. Two reviewers carried out each stage. Discrepancies between reviewers were resolved by discussion. The senior author (MTA) was consulted when an agreement could not be reached.

Data Extraction

Data extraction was performed through an extraction form using Google Forms (Google LLC, Mountain View, CA, USA). The data extraction form included the following variables: the baseline characteristics of the included studies (author’s name, study type, date of publication, country of origin, and study design), the participant’s baseline characteristics (age, gender, and sample size), significant urine biomarkers (pyuria, leukocyte esterase, and nitrite), and type of infective uropathogen obtained on urine culture. In addition, participants' clinical presentation and other congenital anomalies and comorbidities were reported. Two reviewers carried out the data extraction process, and any discrepancies were solved by discussion or consultation with the senior author.

Quality Assessment

The methodological quality of the included studies was evaluated using the National Institute of Health (NIH) tool for each respective study design included [14]. Two reviewers carried out this process. Any discrepancies were resolved by consultation with a senior author.

Data Synthesis

Data from the included studies were retrieved and qualitatively and quantitatively analyzed. The qualitative synthesis included a summary of the results of the included studies (whether pyuria is a sufficient diagnostic tool for UTI) and their correlation with the patient’s baseline data, including age, gender, causative urinary pathogen, urine concentration, presenting symptoms, the interval between the symptoms appearance and sample collection, and the presence of congenital anomalies and comorbidities. Measures of diagnostic accuracy, such as sensitivity and specificity, were also interpreted. Data was represented using frequencies and percentages, and the test’s sensitivity and specificity were assessed using receiver operating characteristic (ROC) curve analysis, and the area under the curve (AUC) was calculated. All calculations were performed using SPSS Statistics version 26.0 (IBM Corp. Released 2019. IBM SPSS Statistics for Windows, Version 26.0. Armonk, NY: IBM Corp.).

Results

Search Results

**Figure 1:**
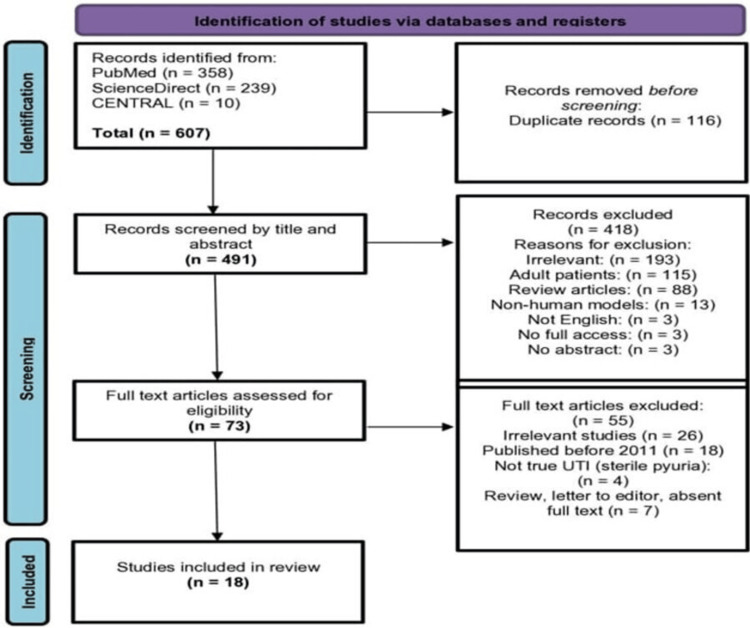
PRISMA chart showing the filtration process PRISMA: Preferred Reporting Items for Systematic Reviews and Meta Analyses

The systematic search yielded a total of 607 papers; 116 duplicates were eliminated, and the remaining 491 papers were screened by title and abstract. Then, 73 potential full texts were assessed, and a total of 18 papers met the predefined criteria. All included studies were observational. A PRISMA diagram illustrating the database search results and the screening process is shown in (Figure 1).

Baseline Characteristics of the Included Studies

A total of 18 papers with 35,110 cases among the studies from nine different countries were included. The mean age of patients was 31.88 months (2.65 years), with an observed predominance of female cases (60%) across the included studies. Detailed demographic data and main outcomes of the studies are summarized in Tables 1-2. The quality of the included studies was assessed using the NIH quality assessment tool, and the results are shown in Table 1.

**Table 1:** Baseline characteristic and diagnostic markers of the included studies *LE: leukocyte esterase, **cath: urethral catheterization, CC: clean catch, SPA: suprapubic aspiration, SUB: sterile urine bag, All: all four methods (cath, CC, SPA, and SUB) UTI: urinary tract infection, NA: not applicable

Paper	Year	Country	Study type	No. of patients	Duration of study	Male (%)	Mean age (months)	Patients with pyuria n (%)	Patients with positive nitrite test N (%)	Patients with positive LE test*	Urine collection method**	Quality	Key findings
Shaikh et al. [13]	2016	USA	Retrospective cross-sectional	1181	2007 to 2013	11%	23	1031 (87%)	N/A	1052	CC, SPA, cath	Good	Children infected with non-*Escherichia coli* strains, including *Pseudomonas aeruginosa*, *Enterococcus* species, or *Klebsiella* species, were significantly less likely to have pyuria and leukocyte esterase than children with *Escherichia coli* strains. It could be concluded that pyuria alone is not an accurate marker for diagnosing UTIs in children. Identifying biomarkers more accurately than pyuria and leukocyte esterase may help reduce over and under-treatment of UTI.
Nadeem et al. [15]	2022	USA	Retrospective cross-sectional	1916	January 2012 to December 2017	33.20%	6.6	1690 (88%)	774	1691	CC, cath	Moderate	This study states that the sensitivity, specificity, and optimal cutoff for pyuria change with urine concentration in children. The highest sensitivity and specificity are associated with lower urine concentrations. Positive leukocyte estrase was a strong predictor of UTI regardless of urine concentration.
Kim et al. [16]; group A	2016	Korea	Retrospective cohort	283	July 2009 to July 2014	73.60%	4.2	279 (99%)	42	251	All	Moderate	This study investigates the absence of pyuria in relation to the duration of fever before hospital visits among febrile infants. Results show that some infants with true UTI might have a normal urinalysis, especially if it was performed too early after the onset of fever. Therefore, UTI cannot be excluded from normal urinalysis in febrile infants. Additionally, circumcision status has factored into the increased prevalence of UTI among males in this sample.
Kim et al. [16]; group B				19	July 2009 to July 2014	63.20%	3.2	0 (0%)	0	0	All	Poor	
Lo et al. [17]	2018	Brazil	Retrospective cross-sectional	501	January 2010 to December 2012	76.90%	1.5	N/A	20	N/A	All	Good	This study describes the clinical, demographic, and laboratory features of UTI in infants ≤3 months old, and it reported that positive nitrite had high specificity but low sensitivity, whereas pyuria had good sensitivity but low specificity.
Fahimi et al. [18]	2021	Iran	Retrospective cross-sectional	104	2012 to 2018	8.66%	47.08	92 (88%)	47	92	All	Good	Acute pyelonephritis is symptomatic and mainly caused by *Escherichia coli* in most children. Pyuria was present in 88.46% of children with acute pyelonephritis in this study.
Yavaş et al. [19]; group A	2021	Turkey	Retrospective cohort	123	December 2015 to June 2016	7.30%	81.7	N/A	37	123	All	Moderate	The study aims to determine the accuracy of the leucocyte esterase test for UTI diagnosis, mainly relying on the absence/ presence of pyuria in LE-positive patients. Children with positive LE tests without pyuria are mostly prepubertal girls, and there is a high rate of vulvovaginitis in these girls. Results also reported that a false positive LE test is significantly associated with phimosis in boys.
Yavaş et al. [19]; group B				164	December 2015 to June 2016	0%	78.5	164 (100%)	1	164	All	Poor	
Shah et al. [20]	2014	USA	Prospective cohort	703	January 2010 to January 2012	23%	10	N/A	N/A	N/A	CC, cath	Good	This study compared manual versus automated UA for urine specimens obtained via catheterization in the pediatric emergency department. Results revealed that enhanced methods exhibited slightly higher sensitivity and specificity rates than automated methods.
Atay and Gökceoğlu [21]	2021	Turkey	Retrospective cross-sectional	361	January 2015 to December 2017	22%	55.8	263 (73%)	158	278	CC, cath	Poor	Pyuria may be absent depending on age, gender, and type of uropathogen. Higher levels of pyuria were observed among females in the studied age group. With respect to uropathogen, pyuria was most associated with the following pathogens in descending order: *Escherichia coli*, *Klebsiella*, *Proteus*, *Enterococcus spp.*, and *Pseudomonas*.
Maduemem et al. [22]	2019	Ireland	Retrospective cohort	262	January 2014 to December 2016	35.88%	69.72	241 (92%)	140	225	All	Moderate	The study discussed the reliability of markers obtained on urine dipstick (LE, nitrite, pyuria) in diagnosing UTI. Dipstick urinalysis alone may not be a completely adequate screening tool for UTI. However, a combination of all three parameters increases the sensitivity of the diagnosis. A strong comparison between a positive LE test and pyuria was reported.
Chaudhari et al. [23]	2018	USA	Retrospective cross-sectional	2554	May 2009 to December 2014	39.60%	6.82	314 (12%)	251	N/A	CC, SUB, cath	Moderate	The diagnostic test characteristics of the urine dipstick and microscopic urinalysis for the diagnosis of pediatric UTI can be affected by urine concentration. Positive likelihood ratios of microscopic pyuria and the presence of leukocyte esterase decreased with increasing urine specific gravity.
Nadeem et al. [24]	2021	USA	Retrospective cross-sectional	24171	January 2012 to December 2017	41.20%	7.3	N/A	24171	N/A	CC, cath	Good	This study reports an inverse relationship between the diagnostic accuracy of pyuria and urine concentration. The optimal cutoff for pyuria changes with urine concentration. Positive LE accuracy, however, was unaffected by urine concentration.
Su et al. [25]	2019	USA	Prospective cohort	50	2004 to 2015	38%	51.6	34 (68%)	N/A	34	All	Good	Pyuria is not a reliable indicator of UTI in patients with neurogenic bladder as it exhibits different patterns among patients from this demographic. Catheterization and surgery were associated with higher levels of pyuria in the absence of any UTI symptoms.
Ünsal et al. [26]	2019	Turkey	Retrospective cross-sectional	705	January 2016 to January 2017	20.30%	65.65	529 (75%)	221	503	All	Good	This study reports the relationship between urinalysis and types of uropathogens. Pyuria was more likely observed in patients infected with *Escherichia coli* and *Proteus* than those infected with *Klebsiella* and *Enterococcus*. Furthermore, pyuria was found to be more common among girls.
Lee et al. [27]	2021	South Korea	Retrospective cohort	145	January 2018 to December 2019	41.37%	8.93	67 (46%)	22	128	SPA, cath	Good	This study determines the diagnostic accuracy of urinary N-acetyl-beta-D-glucosaminidase/creatinine ratio in detecting true pyuria for children with suspected UTI. The NAG/Cr ratio may be a potential indicator for discriminating true pyuria from false pyuria in the pediatric emergency department. Among the true pyuria group, 56 (83.6%) children had a positive urine culture and met the diagnostic criteria for UTI.
Rahman et al. [28]	2011	Pakistan	Prospective cohort	110	April 2008 to August 2008	43%	0.1	35 (32%)	N/A	N/A	Cath	Good	This study aimed to determine the significance of pyuria as a predictor of culture-proven UTI in neonates. More than half the patients with pyuria had no significant growth on urine cultures. Furthermore, pyuria had a significant association with patients with abnormal renal ultrasound findings. Therefore, pyuria can not be relied on to diagnose UTI among this patient demographic.
Gorski et al. [29]	2021	USA	Retrospective cohort	79	November 2015 to February 2017	57.70%	20.5	4 (5%)	2	4	All	Poor	Only two of the six positive urine cultures had pyuria, which grew *Escherichia coli*.
Forster et al. [30]	2017	USA	Retrospective cross-sectional	133	August 2015 to March 2016	52.60%	92.4	N/A	25	N/A	All	Good	The most commonly isolated pathogen was *Escherichia coli*. *Proteus mirabilis* was more likely to be associated with pyuria and leukocyte esterase, whereas *Enterococcus *had lower odds for both*. *Furthermore, a higher proportion of VUR patients were among the pyuria group.
Yodoshi et al. [31]	2019	Japan	Retrospective cross-sectional	1546	January 2011 to December 2015	55%	3	1222 (79%)	NA	N/A	Cath	Good	This study discusses the utility of point-negative gram stain compared with pyuria among children under or at the age of 38 months. Pyuria was inferior to point negative gram stain in the diagnosis of UTI among this age group.

**Table 2:** Types of urinary pathogens and reported congenital anomalies VUR: vesicoureteral reflux, N/A: not applicable

Paper	Number of patients	Anomalies	No. of positive cultures							
			Escherichia coli	Pseudomonas aeruginosa	Staphylococcus saprophyticus	*Enterococcus* species	*Klebsiella* species	*Proteus* species	*Enterobacter* species	Other
Shaikh et al. [13]	1181	N/A	999 (85%)	13 (1%)	27 (2%)	35 (3%)	46 (4%)	31 (3%)	15 (1%)	15 (1%)
Nadeem et al. [15]	1916	N/A	1725 (90%)	0 (0%)	0 (0%)	25 (1%)	94 (5%)	27 (1%)	27 (1%)	21 (1%)
Kim et al. [16]; group A	283	VUR: 67	N/A	N/A	N/A	N/A	N/A	N/A	N/A	N/A
Kim et al. [16]; group B	19	VUR: 12	9 (47%)	0 (0%)	0 (0%)	5 (26%)	5 (26%)	0 (0%)	0 (0%)	1 (5%)
Lo et al. [17]	501	N/A	37 (100%)	0 (0%)	0 (0%)	5 (14%)	12 (32%)	2 (5%)	5 (14%)	4 (11%)
Fahimi et al. [18]	104	Hydronephrosis: 22, cystitis: 9	96 (92%)	N/A	1 (1%)	1 (1%)	1 (1%)	N/A	N/A	2 (2%)
Yavaş et al. [19]; group A	123	Labial synechiae: 3 girls, phimosis: 5 boys	8 (7%)	N/A	N/A	N/A	N/A	N/A	N/A	11 (9%)
Yavaş et al. [19]; group B	164	N/A	67 (41%)	N/A	N/A	N/A	N/A	N/A	N/A	13 (8%)
Shah et al. [20]	703	N/A	38 (5%)	N/A	N/A	5 (1%)	5 (1%)	1 (0%)	N/A	5 (1%)
Atay and Gökceoğlu [21]	361	N/A	308 (85%)	5 (1%)	N/A	8 (2%)	23 (6%)	9 (2%)	N/A	8 (2%)
Maduemem et al. [22]	262	Unspecified structural anomalies: 92	235 (90%)	4 (2%)	4 (2%)	7 (3%)	0 (0%)	6 (2%)	0 (0%)	6 (2%)
Chaudhari et al. [23]	2554	Unspecified structural anomalies: 2	N/A	N/A	N/A	N/A	N/A	N/A	N/A	N/A
Nadeem et al. [24]	24171	N/A	1354 (6%)	N/A	N/A	362 (1%)	1353 (6%)	362 (1%)	386 (2%)	266 (1%)
Su et al. [25]	50	Obstructive uropathy: 50	39 (78%)	39 (78%)	0 (0%)	0 (0%)	19 (38%)	0 (0%)	2 (4%)	15 (30%)
Ünsal et al. [26]	705	N/A	561 (80%)	12 (2%)	0 (0%)	25 (4%)	60 (9%)	35 (5%)	10 (1%)	2 (0%)
Lee et al. [27]	145	N/A	48 (33%)	0 (0%)	0 (0%)	1 (1%)	9 (6%)	1 (1%)	0 (0%)	1 (1%)
Rahman et al. [28]	110	Unspecified structural anomalies: 17	3 (3%)	N/A	N/A	N/A	5 (5%)	N/A	N/A	1 (1%)
Gorski et al. [29]	79	Hydronephrosis: 24, VUR: 4	9 (11%)	1 (1%)	N/A	5 (6%)	4 (5%)	N/A	N/A	2 (3%)
Forster et al. [30]	133	Myelomeningocele: 52, anorectal malformation: 21, tethered cord: 19, cloaca: 6	34 (26%)	3 (2%)	N/A	4 (3%)	9 (7%)	N/A	3 (2%)	5 (4%)
Yodoshi et al. [31]	1546	N/A	126 (8%)	1 (0%)	N/A	11 (1%)	22 (1%)	4 (0%)	4 (0%)	6 (0%)
Total	35110	405	5696 (16%)	78 (0%)	32 (0%)	499 (1%)	1667 (5%)	478 (1%)	452 (1%)	384 (1%)

Association of Causative Uropathogens and Anomalies

The analysis of the microbiological data among studies revealed a total of 9,286 positive urine cultures. Urine was collected using the following methods: CC, urethral catheterization, SPA, and SUB. Gram-negative organisms were dominant over gram-positive organisms. The most frequently isolated pathogen among the urine cultures was* Escherichia coli,* identified in 5,696 (61%) patients, followed by *Klebsiella *species (17%), *Enterococcus *species (5.2%), *Proteus *species (5.1%), *Enterobacter *species (4.8%), *Pseudomonas *species (0.8%), *Staphylococcus saprophyticus *species (0.3%), and others (4.1%). Pyuria was found in 5,965 (64%) of patients with positive urine cultures and was absent in 3,321 (36%) of patients. Among all the studies that reported it, nitrite was positive in 73%, and leukocyte esterase was positive in 0.12%, as illustrated in Table 2.

Congenital anomalies and comorbidities were not reported among all the studied populations. Reported anomalies are presented in Table 2. Of the reported 405 anomalies, vesicoureteral reflux (VUR) was the commonest anomaly reported in (20%, n=83) patients, followed by meningomyelocele in (12%, n=52), obstructive uropathy (12%, n=50), hydronephrosis (11%, n=46), anorectal malformation (5%, n=21), tethered cord (4%, n=19), cystitis (2%, n=9), and cloaca (1%, n=6), in addition to labial synechiae in three girls, phimosis in five boys, and other unspecified structural anomalies (27%, n=111).

Parameters: Sensitivity and Specificity

**Figure 2:**
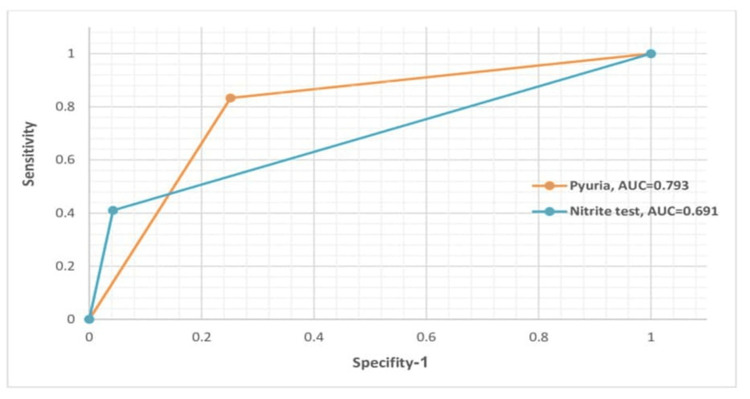
ROC curves illustrating the diagnostic performance of pyuria and nitrite ROC: receiver operating characteristic, AUC: area under the curve

The sensitivity and specificity of pyuria shown in Table 3 exhibited a variable spectrum among the included studies, with sensitivity ranging from 59.10% to 92.20% and specificity ranging from 55.70% to 94.00%, respectively. The nitrite sensitivity ranged from 30.8% to 53%, and specificity ranged from 84.40% to 100%. A ROC for microscopic pyuria and nitrite is shown in Figure 2. The AUC was 0.793 for pyuria and 0.67 for nitrite in the subset of the seven studies that performed such comparisons, highlighting pyuria's potential as a diagnostic tool in pediatric UTI.

**Table 3:** Comparison of pyuria and nitrite test sensitivity and specificity across the studies

Study ID	Sample size	Pyuria		Nitrite test	
		Sensitivity	Specificity	Sensitivity	Specificity
Forster et al. [30]	133	59.10%	76.60%	36.40%	84.40%
Lo et al. [17]	501	87.70%	74.90%	30.80%	100.00%
Maduemem et al. [22]	262	-	-	53%	-
Nadeem et al. [24]	24171	81.87%	91.13%	42.43%	99.77%
Shah et al. (automated) [20]	703	80.00%	90.00%	36.70%	-
Shah et al. (enhanced) [20]		84.00%	94.00%		-
Yavaş et al. [19]	123	92.20%	55.70%	46.80%	99.00%

Discussion

In this systematic review, we aimed to assess the diagnostic value of pyuria in the pediatric population by collecting and analyzing existing cases in the literature where pyuria was reported to be absent in confirmed UTI cases.

The mean age in this review was (31.88) months. A higher percentage of females were affected in this study (60%), consistent with the findings observed in the literature [32,33]. However, UTI is more prevalent among males in the first year of life, specifically in uncircumcised males [16]. This aligns with existing literature that uncircumcised boys during the first six months are 10 to 20 times more likely to develop a UTI. The preputial space is thought to serve as a potential reservoir for bacterial pathogens in boys, responsible for the increased risk of infection [5]. The prevalence increases among girls later in life. In children between the ages of one and five years, the annual incidence of UTI is 0.9% to 1.4% for girls and 0.1% to 0.2% for boys [34]. By the age of 7, approximately 7.8% of girls and 1.7% of boys will have had at least one UTI episode [5]. The increased susceptibility of females to UTI after the first year of life could be attributed to several factors, including the short length of the female urethra, high vaginal pH, and increased bacterial adhesion to the vaginal cells [35,36].

Our findings indicate that the absence of pyuria cannot exclude a UTI in certain clinical scenarios. Many factors significantly influenced pyuria's diagnostic accuracy across the included studies. These factors include the specific type of uropathogen, employed urinalysis techniques, urine concentration, patient demographics (such as age and gender), duration between onset of fever and hospital admission, and underlying congenital anomalies. The prevalence of UTI varies by age, gender, race, circumcision status, and other factors [2,37]. Despite pyuria being emphasized as an important diagnostic marker, according to the American Psychological Association guidelines of 2011, which state the presence of pyuria and growth of a single uropathogen ≥50.000 CFU/mL are diagnostic for UTI [2], our study identified the absence of pyuria among 36% of the UTI confirmed cases. Prevalence of the absence of pyuria has been previously reported in the literature; a study conducted by Hsu et al. reported the absence of pyuria in 44% out of 157 cases [38]. Similarly, a previous meta-analysis by Williams et al. observed the absence of pyuria in 10% of cases [39]. These findings bring to light that cases of UTI without pyuria do exist.

In concordance with existing literature [40,41], *Escherichia coli* was this study's most commonly isolated organism for UTI. Among the included studies, non-*Escherichia coli* pathogens were strongly associated with the absence of pyuria, as reported by Shaikh et al., who found the absence of pyuria in 13% of patients, Unsal et al. in 25% of patients, and Nadeem et al. in 11% of patients [13,15,26]. The same findings were reported by a recent cross-sectional study that discussed pyuria-negative UTI and found that non-*Escherichia coli* pathogens were the strongest risk factor responsible for the absence of pyuria, as non-*Escherichia coli* strains were prevalent in 52% of cases without pyuria and only in 4.5% of cases with pyuria [38].

Furthermore, in a study conducted on 248 pediatric patients with symptomatic UTI, Kenosi et al. reported that all *Escherichia coli*-infected cases exhibited pyuria. In contrast, only 22% of UTI cases caused by non-*Escherichia coli* strains exhibited pyuria [42]. The difference in the pattern of pyuria’s association among *Escherichia coli* and non-*Escherichia coli* strains could be because *Escherichia coli* species are generally more virulent than non-*Escherichia coli* species and, therefore, have the ability to induce a stronger inflammatory response compared to non-*Escherichia coli* strains [43]. Furthermore, the ability of some uropathogens to form intracellular bacterial communities could possibly affect the inflammatory response, leading to the observed variations [44]. Other reasons related to the host genetics, defense mechanisms, and immune response may also influence the levels of pyuria, though they remain poorly understood.

Shorter duration of fever has led to low levels of pyuria. Pyuria was completely absent (0%) in patients who presented to the hospital after a short duration of fever compared to 98.6% of those who were admitted after a longer duration of fever. This observation was noted in another study [38]. This finding was attributed to the absence of inflammation very early in the course of the infection at the time of the initial urinalysis [16]. On a similar note, Gregory and Hey reported that the lack of pyuria in newborns is due to the immature immune response that prevents the formation of a proper inflammatory response during the initial stages of a UTI [45]. A similar mechanism is thought to be responsible for the absence of pyuria in neutropenic patients with UTI, as reported by Klaassen et al., who mentioned the presence of pyuria among 4% of neutropenic children with UTI, compared to 68% of non-neutropenic children. This is attributed to the weakened cellular immune response in neutropenic patients, resulting in a low sensitivity of urinalysis for detecting pyuria in this group of patients [46].

The sensitivity and specificity of pyuria were highly variable among the included studies. The mean sensitivity and specificity of microscopic pyuria were 80.81% and 76.48%, respectively. The urinalysis techniques, particularly enhanced and automated methods, showed varying degrees of sensitivity and specificity for pyuria. Enhanced urinalysis increased the sensitivity and specificity of microscopic pyuria by 4% compared to automated urinalysis. In comparison, automated methods had a sensitivity of 80% and a specificity of 90%, while enhanced methods increased these to 84% and 94%, respectively. Similarly, Shaw et al. reported that enhanced urinalysis had the highest sensitivity (94%) for detecting UTI [47].

Regarding congenital anomalies in this study, VUR was the most commonly reported urinary tract anomaly among our findings, which is consistent with existing studies from the literature [48,49]. There was an association between VUR and higher levels of pyuria and leukocyte esterase [30]. The suggested pathophysiology is that VUR patients have higher levels of IL-8. This chemotactic protein is responsible for neutrophil attraction during an immune response and for the presence of white blood cells in the urine during a UTI [50]. On the contrary, results from an included study reported the absence of pyuria among four patients with VUR [16]. It is noteworthy, however, that this absence could be linked to the timing of urinalysis among these patients, as it was conducted shortly after the onset of fever.

In this review, non-*Escherichia coli* pathogens were significantly associated with underlying renal anomalies compared to *Escherichia coli* strains. This is also reported by Friedman et al., who noted urinary tract anomalies in 18 patients, of whom 14 (77%) were infected by non-*Escherichia coli* pathogens [43]. Moreover, according to Pecurariu et al., VUR was four times more likely in infants infected with *Klebsiella* than in *Escherichia coli* [51]. Data from the literature also indicate that infections with organisms other than *Escherichia coli* have a higher risk for renal scarring, possibly due to underlying congenital anomalies [13].

This study has some limitations. Firstly, there was notable heterogeneity among the included studies. This heterogeneity can hinder the generalization of the study's findings to a larger population. Secondly, the included studies did not agree on a specific cutoff for pyuria, and others did not mention the cutoff for pyuria that was set, which may have influenced the diagnostic accuracy of pyuria. Thirdly, not all included studies reported the congenital anomalies and the associated comorbidities among study populations. Additionally, most of the included studies were retrospective, which may further limit the study design to the variables present in the electronic medical records at the time of the conducted search and make it subject to recall bias. Furthermore, only English studies were included. Lastly, the reliance on observational studies limits the ability to establish causality between pyuria and UTI in pediatric patients.

## Conclusions

UTIs are one of the most common bacterial infections among pediatrics. Clinicians rely on urinalysis for the diagnosis of UTI; however, the results of our study showed that a number of febrile infants and children may have pyuria-negative UTI on initial urinalysis. Certain factors, including age, urinary pathogens, duration of fever prior to urinalysis, and other factors, can lead to this finding. Delayed diagnosis and treatment of UTI could result in complications. Further research and studies are needed to assess the diagnostic performance of pyuria in this age group.
